# Genetic and Metabolic Intraspecific Biodiversity of *Ganoderma lucidum*

**DOI:** 10.1155/2015/726149

**Published:** 2015-03-01

**Authors:** Anna Pawlik, Grzegorz Janusz, Iwona Dębska, Marek Siwulski, Magdalena Frąc, Jerzy Rogalski

**Affiliations:** ^1^Department of Biochemistry, Maria Curie-Skłodowska University, Akademicka 19, 20-033 Lublin, Poland; ^2^Department of Vegetable Crops, Poznań University of Life Sciences, Dąbrowskiego 159, 60-594 Poznań, Poland; ^3^Department of Plant and Soil System, Laboratory of Molecular and Environmental Microbiology, Institute of Agrophysics PAS, Doświadczalna 4, 20-290 Lublin, Poland

## Abstract

Fourteen *Ganoderma lucidum* strains from different geographic regions were identified using ITS region sequencing. Based on the sequences obtained, the genomic relationship between the analyzed strains was determined. All *G. lucidum* strains were also genetically characterized using the AFLP technique. *G. lucidum* strains included in the analysis displayed an AFLP profile similarity level in the range from 9.6 to 33.9%. Biolog FF MicroPlates were applied to obtain data on utilization of 95 carbon sources and mitochondrial activity. The analysis allowed comparison of functional diversity of the fungal strains. The substrate utilization profiles for the isolates tested revealed a broad variability within the analyzed *G. lucidum* species and proved to be a good profiling technology for studying the diversity in fungi. Significant differences have been demonstrated in substrate richness values. Interestingly, the analysis of growth and biomass production also differentiated the strains based on the growth rate on the agar and sawdust substrate. In general, the mycelial growth on the sawdust substrate was more balanced and the fastest fungal growth was observed for GRE3 and FCL192.

## 1. Introduction

*Ganoderma lucidum* (Curtis) P. Karst belongs to the wood decomposing fungi that have been used for medicinal purposes for centuries particularly in China, Japan, and Korea, where it was often associated with health and healing, long life, knowledge, and happiness. The common names for preparations include Lingzhi, Munnertake, Sachitake, Reishi, and Youngzhi [1]. Moreover, numerous publications were produced, indicating that* G. lucidum* may possess antiallergic, antioxidant, analgesic, antifungal, antiinflammatory, antitumor, antiviral, antiparasitic, cardiovascular, antidiabetic, immunomodulating, hepatoprotective, hypotensive and hypertensive, kidney and nerve tonic, and sexual potentiator properties; it also prevents bronchitis and inhibits platelet aggregations and lowers blood pressure, cholesterol, and blood sugar levels [1–11].

In spite of the biotechnological and medicinal importance of* Ganoderma* species, little is known about their current taxonomy and biology. It is evident that the traditional taxonomy of the* Ganoderma* complex based on morphological characters has long been chaotic, thus limiting its uses [12, 13]. Due to the phenotypic plasticity, morphological stasis, and the lack of keys and accessible type specimens, DNA sequence data play a vital role in characterizing the species within the* G. lucidum* complex [13–16]. Phylogenetic studies have proved that extensive convergence or parallelism of morphological characters has occurred during the evolution of* Ganoderma* [17–19]. Because of its specific interhybridization, the genetic background, however, remains relatively unclear and the genetic distance between* G. lucidum* and other* Ganoderma* species remains unevaluated. Consequently, it is difficult to distinguish* Ganoderma* strains, especially closely related strains [20, 21].

The development of tools aimed at the clear-cut and safe identification and assessment of the genetic variability of wild and cultivated strains is thus a fundamental goal of molecular genetics research [22]. Until now, a variety of laboratory-based techniques have been used to study the genetic diversity in* Ganoderma*, for example, isozyme analysis [23], random amplified polymorphism DNA (RAPD) [21, 24, 25], amplified fragment length polymorphism (AFLP) fingerprinting [20, 26], internal transcribed spacers (ITS) 25S ribosomal DNA sequencing technique [13, 14, 16, 27, 28], partial *β*-tubulin gene sequencing [29], PCR-RFLP [20, 30], sequence characterized amplified region (SCAR) [31], intersimple sequence repeat (ISSR) analysis [21], and sequence related amplified polymorphism (SRAP) [32].

However, phylogenies based only on a selected molecular method do not necessarily have the same topology as trees made from morphological or biochemical data [33, 34]. Additionally, the use of genetic techniques alone in fungal diversity studies has sometimes failed [35]. Recently, metabolic profiling technologies have been applied to investigate the taxonomy and metabolic relationships within microorganisms including* Ganoderma* sp. [33, 34, 36]. Bearing this in mind, it seems highly reasonable and fully justified to use a comprehensive approach in the research concerning identification and differentiation of fungal species taking into account molecular, morphological, physiological, and metabolic data [12].

Therefore, the aim of the present study was to determine the intraspecific diversity of medicinally important fungus* Ganoderma lucidum* based on a complex survey using genetic and biochemical profiling tools. In addition, we investigate the usefulness of these methods for identification and establishing the genomic and metabolic relationships between* Ganoderma lucidum* strains.

## 2. Materials and Methods

### 2.1. Fungal Strains and Cultivation

*Ganoderma lucidum* strains (Table 1) were obtained from the Department of Vegetable Crops, University of Life Sciences, Poznan, Poland (ULSP), and Agriculture University, Tokyo, Japan (FCTUA). The stock culture of fungal strains was maintained on GPY slants (glucose 1 g/L, peptone 0.5 g/L, yeast extract 0.1 g/L, agar 20 g/L). The slants were inoculated with mycelia and incubated at 25°C for 7 days and then used for seed culture inoculation. The mycelia of* G. lucidum* strains were transferred into a 100 mL Erlenmayer flask containing 40 mL stationary liquid Lindeberg-Holm (LH) medium [38] by punching out about 5 mm^2^ of the slants with a sterilized cutter. The seeds were cultivated for 14 days at 25°C. Broth cultures were then harvested by centrifugation at 10,000 ×g for 10 minutes and used for DNA extraction.

### 2.2. PCR Amplification and Sequencing of the Fungal ITS Region

The mycelia from 40 mL liquid cultures were used for DNA extraction according to the method of Borges et al. [39]. The purity and quantity of the DNA samples were evaluated using an ND-1000 spectrophotometer (Thermo Scientific, Palm Beach, FL, USA). PCRs were performed using Sigma RedTaq in a T-personal thermal cycler (Biometra, Goettingen, Germany). To confirm the genetic identity of the fungus, the ITS region in the nuclear ribosomal repeat unit was determined by direct sequencing of the PCR products amplified with ITS1-ITS4 primers as described previously [40, 41]. Automatic sequencing was performed using a BigDye Terminator Cycle Sequencing Kit and an ABI PRISM 310 or ABI PRISM 3730 XL sequencer (Applied Biosystem).

### 2.3. AFLP Analysis

The AFLP reactions were performed according to Vos et al. [42] with modifications as described below. Adapters and primers were synthesized by GensetOligos, France, and IBB PAN, Poland.

#### 2.3.1. Restriction-Ligation

The genomic DNA (1 *μ*g) was digested in the final volume of 30 *μ*L with 20 U of the* Pst*I restriction enzyme (Fermentas, Lithuania) for 18 hours at 37°C. The quality and quantity of the digested product were examined by 0.7% gel electrophoresis, stained with ethidium bromide, and visualized under UV fluorescence as a smear across bromophenol blue.

The double-stranded* Pst*I oligonucleotide adapters were formed in a total volume of 10 *μ*L by incubating 10 *μ*M* Pst*I_AF and 10 *μ*M* Pst*I_AR adapters at 95°C for 10 minutes, following 30 minutes at room temperature.

The ligation solution containing the double-stranded adapters (10 *μ*L), DNA digested with* Pst*I (850 ng), 5 U T4 DNA polymerase (Fermentas, Lithuania), and 1 X T4 ligase buffer (40 mM Tris-HCl, 10 mM MgCl_2_, 10 mM DTT, and 0.5 mM ATP pH 7.8) was incubated for 4 hours at 37°C (25 *μ*L final volume). Ligated DNA was then precipitated with a mixture of 3 M sodium acetate, pH 5.5 and ice cold 96% ethanol (1 : 25) at −18°C for 30 minutes to remove unbound adapters. DNA was harvested by centrifugation (14,000 rpm, 4°C, 20  minutes) and dried in a vacuum centrifuge. The debris of DNA was dissolved in 50 *μ*L of sterile water and used as a template in the amplification reaction.

#### 2.3.2. Nonselective PCR Amplification

Nonselective PCR was performed to check digestion and ligation reactions. PCR was carried out in 20 *μ*L volume containing 5 *μ*L of ligated with double-stranded adapters and purified DNA, 0.2 mM of each dNTP, 1.5 mM MgCl_2_, 0.4 U Taq DNA polymerase LC, recombinant (Fermentas, Lithuania), 1 X PCR buffer (75 mM Tris-HCl pH 8.8, 20 mM (NH_4_)_2_SO_4_, 0.01% Tween 20), and 750 nM* Pst*I_AF primer. Amplifications were carried out in a T-personal thermal cycler (Biometra, Germany) with the conditions as follows: 95°C for 2 min 30 s followed by 45 cycles of 45 s at 94°C, 45 s at 54°C, and 45 s at 72°C. The final cycle was followed by an additional 10 min at 72°C.

#### 2.3.3. Selective PCR Amplification

PCRs were performed in a 50 *μ*L total volume which consisted of 1 X PCR reaction buffer (Fermentas, Lithuania), 2.5 mM MgCl_2_, 0.2 mM of each dNTP, 1 U of Taq DNA Polymerase LC, recombinant (Fermentas, Lithuania), 10 pmol of each primer, and 0.5 *μ*L of targeted digested and ligated genomic DNA. All amplification reactions were performed in a T-personal thermal cycler (Biometra, Germany) with the conditions as follows: 94°C for 2 min 30 s followed by seven cycles of amplification, with annealing temperature decreasing 1°C/cycle: 94°C for 30 s, first annealing for 30 s at 67–61°C or 60–54°C (annealing temperature depends on primer Tm), 72°C for 30 s, and next 33 amplification cycles of 94°C for 45 s, 61°C or 54°C (annealing temperature depends on primer Tm) for 45 s and 72°C for 45 s. The final cycle was followed by an additional 7 min at 72°C. The PCR products were stored at 4°C until further analysis. The adapters and primers employed for AFLP are shown in Table 2.

#### 2.3.4. Electrophoresis and Imaging

For amplicon separation, a Microchip Electrophoresis System for DNA/RNA analysis MCE-202 MultiNA (Shimadzu, Japan) and a DNA-2500 reagent kit were applied. A 5 *μ*L aliquot of the PCR reaction mixture was combined with 1 *μ*L of separation buffer and fluorescent dye SYBR Gold in a 96-well plate. The PCRs were run at 1.5 kV using a WE-C microchip according to the manufacturer's protocol.

### 2.4. Analysis of the Fungal Metabolic Profile Using Biolog FF MicroPlates

The global phenotypes and utilization of particular nutrients by each of the* G. lucidum* strains based on 95 low molecular weight carbon sources were evaluated using the Biolog FF MicroPlate (Biolog, Inc., Hayward, CA). The inoculation procedure was based on the original FF MicroPlate (Biolog Inc., Hayward, CA) technique (manufacturer's supplied protocol) and the protocol was modified by Frąc [43]. For inoculum preparation, mycelia of each strain were obtained by cultivation on 2% MEA plates in the dark at 27°C for 14 days. The mycelia were thoroughly macerated using a spatula or a battery-operated mini-grinder to fragment the mycelia. The suspension of the mycelia in inoculating fluid (FF-IF, Biolog) was adjusted to 75% of transmittance as measured by a turbidimeter (Biolog). 100 *μ*L of the above-mentioned mycelial suspension was added to each well and the inoculated microplates were incubated at 27°C in the OmniLog ID System (Biolog, Inc., Hayward, CA). The optical density was determined using a Biolog microplate reader for each plate at 24 h intervals over the period of 336 h at 490 nm (mitochondrial activity) and 750 nm (mycelial growth), in triplicates. The most consistent readings came from the 9-day old Biolog plates and these were used in the analyses.

### 2.5. Growth and Biomass Production

In the first experiment* Ganoderma lucidum* mycelia growth was compared on PDA (Oxoid, England), MEA (Merck, Germany) and wheat agar media. Wheat agar medium was prepared on an extract of wheat grain. The extract was obtained by boiling 125 g of grain in 1 dm^3^ of distilled water for 30 minutes. After the wheat grain was strained on a cedar, 3 g of glucose and 22 g of agar were added, and distilled water was added to complete the volume of 1 dm^3^. The incubation was carried out at the temperature of 25°C for 6 days.

In the second experiment, the growth of* Ganoderma lucidum* mycelia on sawdust substrate was compared. A mixture of beech and poplar sawdust (1 : 1) supplemented with wheat bran in the amount of 20% in relation to the substrate dry matter was wetted with distilled water to the moisture content of 65% and placed in glass test tubes (2 × 16 cm). Next, the culture medium was sterilized at the temperature of 121°C for 30 minutes and after cooling down to the temperature of 21°C the substrate was inoculated with the mycelium (mycelium on the wheat grains). The mycelium was placed on the surface of the substrate in a layer of 1 cm. The inoculated test tubes were incubated at 25°C and 80–85% RH for 10 days.

### 2.6. Data Treatment

#### 2.6.1. Bioinformatic Tools in ITS Analysis

Data from ITS sequencing was analyzed with Lasergene v.8.0 software (DNASTAR, Inc). Database searches were performed with the BLAST and FASTA programs at the National Centre for Biotechnology Information (Bethesda, MD, USA) and European Bioinformatic Institute (Hinxton, UK). The DNA sequence multiple alignments were performed with the Clustal-W algorithm [44]. Phylogenetic tree visualization was performed using the TreeView applet [45].

#### 2.6.2. Data Analysis of AFLP Results

Gel images/pherograms were visualized and analyzed using MultiNA Control & Viewer Software (Shimadzu, Japan). Presence or absence of the band between 100 and 2500 bp was regarded as a single trait and values 1 or 0 were assigned respectively. This binary information was used to calculate Jaccard's pairwise similarity coefficients as implemented in the program FreeTree version 0.9.1.50 [46]. On the basis of the DNA band patterns Dice's similarity was determined as in Nei and Li [47] and cluster analysis was performed. The UPGMA (unweighted pair-group method with arithmetic averages) method was used for clustering, employing NTSYSpc software version 2.01. (Exeter Software Co., New York).

#### 2.6.3. Biolog Data Treatment

Data from all experiments were combined in a single matrix and analyzed with the STATISTICA 10.0 (StatSoft, Inc., Tulsa, OK) software package. All data were subjected to descriptive statistical evaluations (mean, minimum, maximum, and standard deviation values) and checked for outliers. The average well color developments (AWCDs) of the different replicates were calculated, where AWCD equals the sum of the difference between the OD of the blank well (water) and substrate wells divided by 95 (the number of substrate wells in the FF plates) developed by the fungus after 216 h of incubation. Functional diversity was measured as substrate richness. The number of different substrates utilized by the strain (counting all positive OD readings) was calculated. Cluster analysis [48, 49] was used to detect groups in the data set. In most cases, the cluster-joining analysis was made with Euclidian distance and complete linkages as the amalgamation rule, that is, distances between clusters were determined by the greatest distance between any two objects in the different clusters. One-way or main-effect analyses of variance ANOVAs (confidence interval 95%) were performed to compare the growth of selected strains on individual carbon sources. ANOVA was followed by a post hoc analysis using the Tukey's HSD (honestly significant difference) *t*-test. The summed data matrixes also were evaluated following multidimensional scaling to detect additional relationships between variables.

#### 2.6.4. Statistical Analysis of the Growth Tests

Both experiments were established in a completely randomized design in 5 replications. The results were analyzed using variance for two-factorial experiments at the level of significance of *α* = 0.05 (Newman-Keuls test).

## 3. Results

### 3.1. Fungal ITS Region Analysis

One product was obtained from PCR with ITS1-ITS4 primers and followed by direct sequencing. The complete sequences of these products showed slight differences in the length polymorphism between the strains, ranging from 636 (strain FCL188, FCL191, FCL192, FCL193, FCL194, FCL195, FCL196, and FCL265) to 643 bp (strain GL04) and revealed over 99% identity to* Ganoderma lucidum*, as shown in the BLAST analysis. The GenBank accession numbers assigned to the nucleotide sequences determined in this study are presented in Table 1.

**Figure 1:**
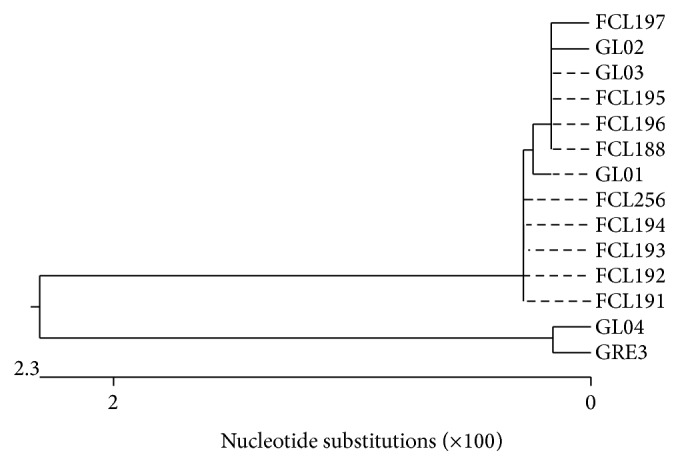
Phylogenetic tree based on ITS region sequences for the 14* Ganoderma lucidum* strains.

The alignments of the obtained* Ganoderma lucidum* ITS sequences indicated similarities between the strains ranging from 95.3 to 100% (data not shown). Phylogenetic analysis of these sequences produced two main clusters: one including strains GRE3 and GL04 and another containing the remaining 12 strains (Figure 1).

### 3.2. AFLP Fingerprints

The rare cutting restriction endonuclease* Pst*I and 4 primers listed in Table 2 were used separately in selective DNA amplification of 14* G. lucidum* strains in the AFLP fingerprinting analysis.

The smears obtained in the nonselective PCR amplification (data not shown) proved efficient degradation of DNA by* Pst*I endonuclease. In the selective amplification reactions, all primers successfully amplified AFLP bands in all the fungi studied. Each of the four primers generated a fingerprint pattern markedly distinct from those of the other primers, even when the primers differed in only one selective nucleotide in the extension. A total of one to three selective bases were found to provide a sufficient complex pattern for the DNA polymorphism analysis. Although a variable number of amplified bands were obtained in the PCR reaction with each primer, all of them generated polymorphic and unambiguously scored fragments.

The AFLP method applied has provided characteristic genomic markers to differentiate among the* G. lucidum* strains. High resolution and high reproducibility of the obtained biological data were achieved by application of an automated electrophoresis system (Shimadzu, Japan). Selective primers generated a total of 436 robust and reliable fragments, including 112 monomorphic (25.7%) and 324 polymorphic (74.3%) ones. The large number of bands obtained in the PCR reaction with all the primers demonstrates that the AFLP analysis is a robust and efficient method for detecting genomic differences among the analyzed strains. The primers differed in their ability to detect polymorphism among the strains; the number of scorable amplicons produced high variation and ranged from 1 to 19 with an average of 109 per primer combination.

A binary matrix was used to compute similarities among the* Ganoderma* strains (Table 3). Average Jaccard's similarity coefficient [50] among the studied strains was low, that is, 0.312. The highest similarity coefficient, 0.735, was found between two Japanese strains (FCL191 versus FCL193), the lowest (0.057) between an isolate from Poland (GRE3) and one from Japan (FCL192).

**Figure 2:**
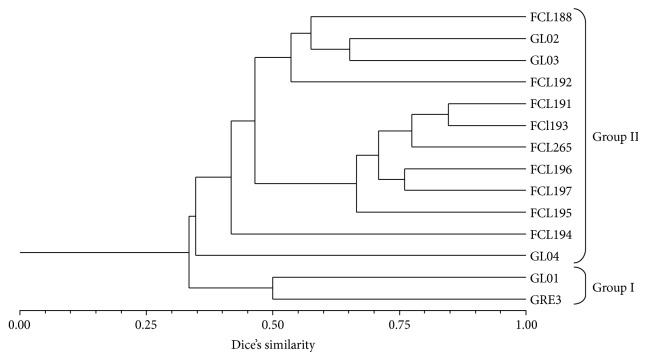
Dendrogram of the 14* G. lucidum* strains generated by UPGMA clustering based on Nei and Li's genetic distance (1979).

The results of the AFLP analysis presented on the dendrogram constructed with the UPGMA method (Figure 2) failed to identify any spatial clustering among the different geographic regions. The analysis showed that the strains of* G. lucidum* separated into two main clusters. Out of the 14 fungi analyzed in this study, 2 were classified as group I and 12 as group II at the DNA profile similarity of 33%. The first group comprised only GL01 from Japan and GRE3 from Poland.* Ganoderma* GL02, GL03 (Poland), FCL256 (Canada), FCL188, FCL192, FCL191, FCL193, FCL196, FCL197, FCL195, FCL194 (Japan), and GL04 originating from China were clustered in the second group. The analysis revealed existence of subgroups within group II and a clear separation of the GL04 strain from the remaining cluster structure. The highest genetic similarity, 0.85, was exhibited by* Ganoderma* FCL191 and FCL193. Strain GL04 classified outside any subgroup and the other* G. lucidum* strains included in the analysis displayed the AFLP profile similarity level in the range from 9.6 to 33.9%.

### 3.3. *G. lucidum* Metabolic Diversity Using the Biolog System

**Figure 3:**
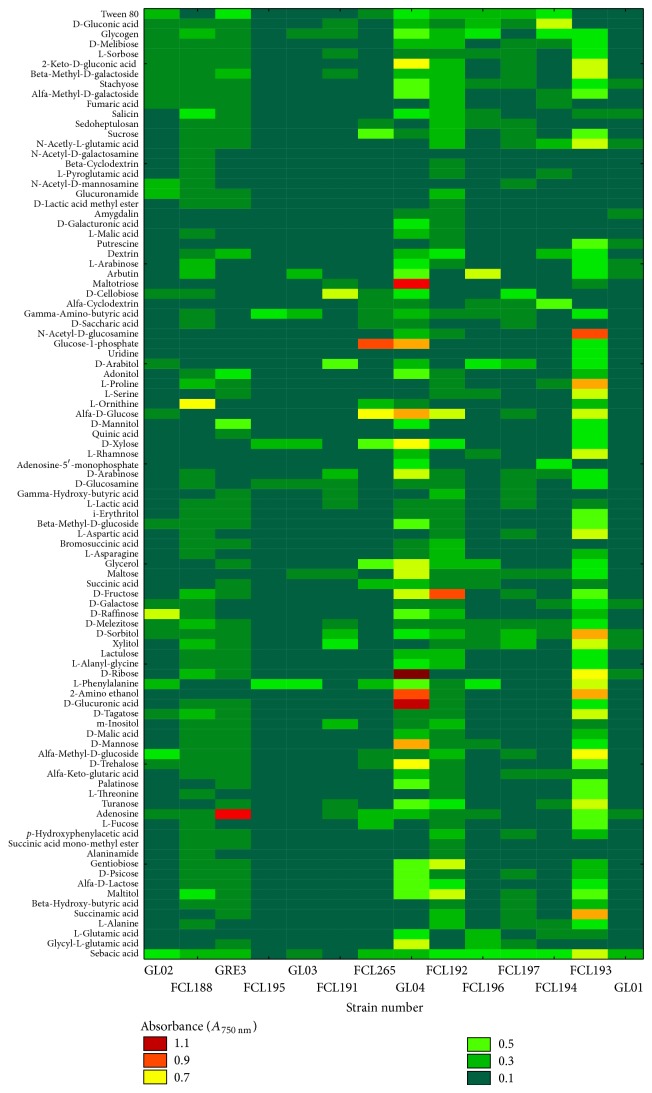
Phenotype profiles of* G. lucidum*. Color scale into the heat maps indicates the growth of the organism in particular substrate during 216 hours of incubation.

**Figure 4:**
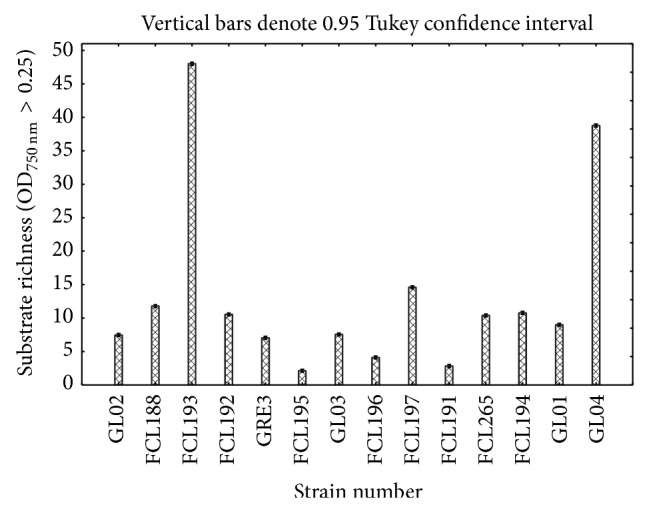
Functional diversity of the analyzed* G. lucidum* strains (substrate richness).

**Figure 5:**
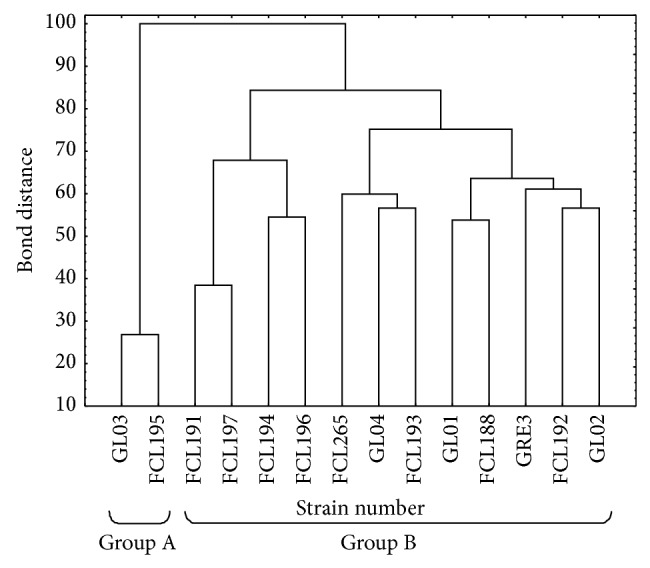
Cluster analysis-based dendrogram showing correlation between the* Ganoderma lucidum* strains in relation to utilization of C-sources from the FF MicroPlate.

Using the FF MicroPlates average well color development and mycelial density analyses allowed for comparison of functional diversity of the 14* Ganoderma lucidum* strains. The substrate utilization profiles for the isolates tested revealed a broad variability (Figure 3). Significant differences (up to 10 times) were demonstrated in the substrate richness values (Figure 4). Strains FCL193 and GL04 showed the highest catabolic activities, which was reflected by their capabilities to decompose 49/95 (51.58%) and 41/95 (43.15%) of the total number of the substrates tested, respectively. In turn, strains FCL195, FCL191, and FCL196 were able to assimilate only 3 to 5 C-sources; that is, 4.2% of the substrates on average. There is no clear correlation in metabolic preferences of the analysed* G. lucidum* strains to a particular group of substrates. However, most fungal strains were easily capable of carbohydrates and carboxylic and acetic acids utilization (Figure 4). N-acetyl-D-glucosamine was utilized only by* Ganoderma* FCL188, whereas uridine only by strain FCL193. Only one carbon source, sebacic acid, was used most universally. Only two strains (FCL195 and FCL191) were unable to utilize this compound. At an 84% similarity level, all the* Ganoderma* isolates were grouped into two major groups (A and B) (Figure 5). In general, the strains from group A used fewer substrates, 8/95 (8.42%), than the isolates from group B. The first cluster (A) comprises only two slowly metabolizing strains: GL03 and FCL195. The second group (B) includes the remaining twelve strains arranged in subclusters (Figure 5). It is worth noticing that the rapidly metabolizing* G. lucidum* GL04 and FCL193 were clustered together at a bond distance of 56%.

### 3.4. Analysis of Growth and Biomass Production

**Figure 6:**
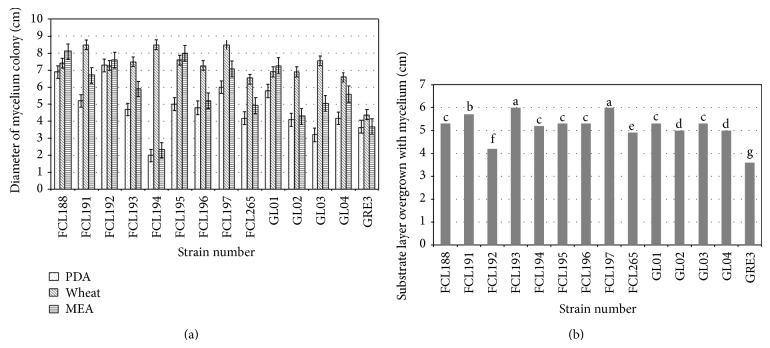
Mycelium growth of the examined* G. lucidum* strains on the different agar media (a) and on the sawdust substrate (b).

The response of the examined* G. lucidum* strains to the kind of agar substrate varied (Figure 6(a)). The fastest growth of the mycelium of the majority of strains was determined on the wheat-agar substrate. Only in the case of the FCL188 strain, the mycelium was found to grow best on the MEA substrate, whereas in the case of the FCL192, FCL195, and GL02 strains, mycelium growth on the wheat-agar and MEA substrates was similar. It was observed that the examined* G. lucidum* strains were found to grow slowest on the PDA substrate. On the other hand, the mycelium growth of the FCL192 strain on the three examined substrates was similar, whereas the mycelia of the FCL194, FCL196, FCL265, GL02 and GRE3 strains exhibited similar growth on the PDA and MEA substrates.

The growth of the examined* G. lucidum* strains on the sawdust substrate was varied (Figure 6(b)). The fastest growth was reported in the case of the mycelia of FCL193 and FCL197 strains followed by the mycelium of the FCL191 strain. Slower growth was observed for the following six strains: FCL188, FCL194, FCL195, FCL196, GL01, and GL03. Even slower mycelium growth was found in the GL02 and GL04 strains followed by the FCL256 and FCL192 strains. The mycelium of the GRE03 strain was characterised by the slowest growth.

## 4. Discussion

*G. lucidum* has been used as a medicinal mushroom in Traditional Chinese Medicine (TCM) for more than 2,000 years [51], thus making it one of the oldest mushrooms known to have been used medicinally. As misidentification of* Ganoderma* strains may hinder strategies for drug discovery [12, 13] and create complications for publications, patents, and products [52], the correct identification of commercial and research-oriented* Ganoderma* strains especially those labelled* G. lucidum*, is obviously important. Recently, the ITS region has been identified as a standard barcode marker for fungi [53]. The genetic identification of the* Ganoderma* taxa based on ITS sequencing has also been widely studied [29, 54]. In the present work, sequencing of the ITS region, including the intervening 5.8S gene, from the total DNA using primers ITS1 and ITS4 successfully allowed identification of* Ganoderma lucidum* strains from different geographic regions (Table 1). The sequenced ITS region varied slightly in length up to 7 bp. There are also publications relating to the use of the ITS region to study the sequence variation (biodiversity) in fungi, including* Ganoderma* species [14, 20, 27, 28, 54]. Even though ITS sequencing is used for the identification and differentiation of microorganisms at the species level alignment of ITS sequences performed here resulted in construction of a phylogenetic tree consisting of two main clusters (Figure 1). Although there was no clear geographical correlation among the strains, all the* Ganoderma* strains originating from Japan were clustered together and the only strain derived from China (GL04) showed the greatest similarity with GRE3 (Poland).

AFLP is a PCR-based technique that can be applied to DNAs of many sources and complexity, and it has been widely reported to be suitable for identification and differentiation of microorganisms at the intraspecies level as well as for determining their genomic relationships [22, 55, 56]. The use of a simplified AFLP protocol as described elsewhere [55, 57] and an automated microchip electrophoresis system produced more reliable and reproducible DNA bands in gel images/pherograms. Due to the fact that the AFLP technique is capable of simultaneous screening many different DNA regions distributed randomly throughout the genome [56], it was possible to obtain a unique genetic fingerprint of the whole microorganism in the case of all the 14* Ganoderma lucidum* strains. Based on the AFLP profiles and UPGMA clustering, the* Ganoderma* isolates were grouped in 2 main clusters (Figure 2). Almost all (except the GL01) the Japanese* G. lucidum* strains were grouped together. A similar relationship was observed for the Polish GL02 and GL03 strains.

In general, there was a compliance of the overall tree topology and a considerable consistency between the results of grouping (Figures 1 and 2) obtained in the AFLP and ITS analysis (e.g., the placement of FCL265 (Canada) among the Japanese strains and GL04 (China) on the outskirt or grouping GL02 and GL03 (Poland) together in the dendrograms). The source of the existing differences can be assigned to the fact that the AFLP technique amplifies randomly the whole genomes while the ITS method is based on ca. 650 bp conserved DNA fragments and usually is applied for discrimination strains at species level [40–42]. Moreover, the cluster analysis of the* G. lucidum* ITS sequences and AFLP profiles also revealed differences in the geographical grouping between the analyzed strains. Although some strains clustered together, others did not, for example, GRE3 is not grouped with the other Polish strains (GL02 and GL03), as shown in Figures 1 and 2. Recent study on 32 collections belonging to the* G. lucidum* complex from Asia, Europe and North America, in terms of their morphology and phylogeny as derived from analysis of four loci (ITS,* tef1*,*α rpb1*, and* rpb2*), proved morphological similarity of the analyzed species but, with respect to phylogeny, all formed at least three lineages that cannot be defined by their geographic distributions [12]. As all other cultivated fungi, the cultivated lines of* Ganoderma* sp. can undergo a drastic loss of diversity resulting from man's selection during 2,000 years as well and outcrossing of the isolates over generations [58, 59]. It has already been proved that fungi growing in the same habitat (laboratory conditions) may undergo loss of genetic diversity, which consequently means losing some genes [22, 60]. Thus, in light of these data, the boundaries of the geographical occurrence of fungal (cultivated) strains are blurred and a simple conclusion concerning fungal origin cannot always be made. However, attention must be paid to correct identification of fungi as* Ganoderma lucidum* and other* Ganoderma* genus were often misnamed [13, 16, 54]. Molecular phylogenetic analyses based on the ITS and 25S ribosomal DNA sequences indicated that most of the collections named as “*G*.* lucidum*” in East Asia were not conspecific with* G. lucidum *found in Europe [16]. It is suggested that* G. lucidum* sensu stricto may be restricted to Europe and that “*G. lucidum*” in Asia consists of at least two distinct species, one represented by material from mainland China and one by tropical Asian collections [27].

Metabolic characters are becoming increasingly important in fungal taxonomic studies [33, 36, 61]. Since Simonić et al. [36] have recently shown possibility of using the enzyme production ability as a taxonomic character for separation of strains within* G. lucidum sensu lato*, metabolic features should be treated as an important part of the process of fungal identification and diversification. On the other hand, it was observed that the enzyme synthesis by fungal isolates belonging to the same species may exhibit a significant/drastic variation and is affected by culture conditions [62, 63]. In this study, the Biolog FF MicroPlates analysis was performed to assess the ability of* G. lucidum* to decompose various substrates. Using this method, the metabolic diversity of* Trichoderma* sp. isolated in South-East Asia and from Colombia has already been determined [33, 35]; however, it should be noticed that moulds adapt more easily to changing environmental factors than white-rot fungi. To our knowledge, this is one of the first such complex surveys on the metabolic diversity of medicinally important fungi. Until recently, the metabolic diversity of* Ganoderma lucidum* has been investigated with chromatographic techniques [64, 65] and based on the abilities of synthesis of specific enzymes [36]. A closer examination of the Biolog data revealed interesting differences in the metabolic properties of the analyzed* Ganoderma* strains. The grouping analysis showed separation of the strains into two main clusters and failed to identify any spatial clustering among the different geographical regions (Figure 5). The results of phenotype grouping (Figure 5) placed slowly metabolizing strains (GL03 and FCL195) together (group A), while GL04 and FCL193, which were able to assimilate 41/95 and 49/95 carbon sources, respectively and were characterized by the highest catabolic activities, occupied the same subgroup in cluster B. Only one carbon source, sebacic acid, was used most universally. It was proposed that the specific dicarboxylic acids are potential metabolites participating in the control of iron redox reactions and charge transfer complexes formation from oxidized lignin fragments. The suppression of the cellulolytic active oxygen species by these metabolites contribute to the selective lignin-degradation with a minimum loss of cellulose [66]. The Biolog experiments have demonstrated a great variability within the analyzed* G. lucidum* species and have proved to be a good profiling technology for studying the diversity in fungi. It should be noted that the ability of some* Ganoderma* strains to degrade a few C-sources may be a result of nutrient specialization to the specific tree species/kind or passaging through a few culture media in a laboratory habitat, resulting in loss of genes. It is also worth mentioning that we do not have knowledge about the physiological condition of the strains and their age.

As compared with the Biolog results, the analysis of growth and biomass production did not differentiate the strains significantly. However, a greater level of metabolic versatility was observed within a specific strain, based on the culture medium that was used. Zakaria et al. [67] have reported that* Ganoderma* species grown on the same host, cluster together in RAPD and PCR-RFLP analyzes. The selection of culture media was made due to the possibility of application thereof in industrial process of fungus cultivation (wheat) and using substrates that were not represented in the Biolog MicroPlate. Interestingly, the strains differed in the growth rate on the agar substrate (FCL194, GL03 versus FCL192 or GRE3), as shown in Figure 6(a). The fungal growth on the sawdust substrate was more balanced, whereby the fastest mycelial growth was observed for GRE3 and FCL192 (Figure 6(b)), which differed greatly when cultured on agar media (Figure 6(a)). The nutrient medium is known to be a major factor that influences fungal growth [68, 69]. It seems surprising that GL04, utilizing maltose, glycerol, and dextrin (Figure 3)—the components of the MEA medium, is characterized by an average rate of growth when cultured on malt extract agar (Figure 6(a)), compared with the relatively fast growth of FCL195, which proved to be the slowest metabolizing strain in the Biolog experiments. It could be explained by the fact that* G. lucidum* presents a worldwide distributed species-complex in which each strain is characterized by different physiological state and metabolic demands [62]. In general, all the strains cultured on unidentified natural substrates (wheat extract and beech and poplar sawdust) were characterized by a more balanced growth rate, and a lower level of diversity was observed. This feature could be treated as a kind of evolutionary adaptation to environmental habitats.* Ganoderma lucidum* is a rotting fungus decomposing raw plant material, which consists of complex polymers and other compounds [70]. The information about fungal geographic location and host's nature might be used for the analysis of genetic diversity, genetic preservation and identification of* Ganoderma* species [21].

## 5. Conclusions

Summarizing, this is the first report on the genetic and metabolic diversity of cultivated medicinal* Ganoderma lucidum* strains of different geographical origin. It is evident that the Biolog groupings do not correlate with the grouping based on the ITS sequences and AFLP profiles. Additionally, taking into consideration the loss of diversity resulting from man's selection and outcrossing of the isolates over generations, one cannot clearly conclude about the geographical origin of the fungi. Due to the vegetative method of fungal propagation in a laboratory, the fungal age and physiological state cannot be inferred. Despite passing by different media in laboratory conditions the loss of genes is still observed, which may be a natural adaptation/specialization to decompose single wood species during vegetative growth and aging. In contrast to sexual propagation in a natural habitat, mixing of genetic material may occur as strain rejuvenation. However, there is lack of understanding of aging in white rot fungi and methods allowing assessment of their age. As shown, the fungal variability is a complex issue and a cautious approach is needed. Further large-scale studies are required, especially bearing in mind that metabolic differences among strains are greater than may be expected based on a genome* in silico* research.

## Figures and Tables

**Table 1:** List of fungal strains used in this study.

Strain number in FCL^a^	Strain name	Strain source/other collection^b^	Geographical origin	GeneBank Accession/Reference
FCL188	*Ganoderma lucidum *	FCTUA 32	Japan	JN008869
FCL191	*Ganoderma lucidum *	FCTUA 35	Japan	JQ627589
FCL192	*Ganoderma lucidum *	FCTUA 36	Japan	JN222423
FCL193	*Ganoderma lucidum *	FCTUA 37	Japan	JQ627590
FCL194	*Ganoderma lucidum *	FCTUA38	Japan	JN008870
FCL195	*Ganoderma lucidum *	FCTUA 39	Japan	JN008871
FCL196	*Ganoderma lucidum *	FCTUA40	Japan	JN222424
FCL197	*Ganoderma lucidum *	FCTUA 41	Japan	JN008872
FCL265	*Ganoderma lucidum *	ULSP Canada LZ	Canada	JN222405
GL01	*Ganoderma lucidum *	ULSP GL01	Japan	JN222421 [37]
GL02	*Ganoderma lucidum *	ULSP GL02	Poland	JN222425 [37]
GL03	*Ganoderma lucidum *	ULSP GL03	Poland	JN222426
GL04	*Ganoderma lucidum *	ULSP GL04	China	JN222422 [37]
GRE3	*Ganoderma lucidum *	ULSP GRE3	Poland	JQ627587

^a^FCL: Fungal Collection of Lublin, Department of Biochemistry, Maria Curie-Sklodowska University, Lublin, Poland.

^b^FCTUA: Forest Products Chemistry Laboratory, Agriculture University, Tokyo, Japan; ULSP, Department of Vegetable Crops, University of Life Sciences, Poznan, Poland.

**Table 2:** List of oligonucleotide primers and adapters.

	Adaptor name	Adaptor sequence 5′-3′	Melting temperature [°C]
1	*Pst*I_AF	C T C G T A G A C T G C G T A C A T G C A	51
2	*Pst*I_AR	T G T A C G C A G T C T A C	42

	Primer name	Primer sequence 5′-3′	Melting temperature [°C]

1	*Pst*I_G	G A C T G C G T A C A T G C A G G	49.5
2	*Pst*I_GC	G A C T G C G T A C A T G C A G G C	52.6
3	*Pst*I_GCG	G A C T G C G T A C A T G C A GG C G	55.41
4	*Pst*I_ACG	G A C T G C G T A C A T G C A G A C G	53.25

**Table 3:** Jaccard's pairwise similarities between the analyzed *G. lucidum* strains calculated on the basis of 324 polymorphic bands.

	Fungal strain	1	2	3	4	5	6	7	8	9	10	11	12	13	14
1	FCL188	1.000													
2	FCL191	0.354	1.000												
3	FCL192	0.300	0.224	1.000											
4	FCL193	0.362	0.735	0.255	1.000										
5	FCL194	0.282	0.246	0.250	0.296	1.000									
6	FCL195	0.366	0.510	0.244	0.551	0.216	1.000								
7	FCL196	0.357	0.472	0.182	0.481	0.189	0.500	1.000							
8	FCL197	0.349	0.580	0.262	0.625	0.280	0.458	0.614	1.000						
9	FCL265	0.294	0.611	0.245	0.654	0.220	0.472	0.549	0.600	1.000					
10	GL01	0.200	0.271	0.225	0.276	0.200	0.320	0.264	0.236	0.267	1.000				
11	GL02	0.433	0.362	0.462	0.400	0.324	0.341	0.273	0.357	0.300	0.262	1.000			
12	GL03	0.375	0.354	0.345	0.333	0.351	0.366	0.239	0.261	0.245	0.350	0.483	1.000		
13	GL04	0.127	0.339	0.096	0.281	0.193	0.233	0.172	0.246	0.254	0.180	0.220	0.192	1.000	
14	GRE3	0.135	0.189	0.057	0.170	0.143	0.174	0.122	0.120	0.143	0.333	0.171	0.200	0.224	1.000
